# Application of laparoscopic backtracking full-thickness continuous everting suture for non-AOSC choledocholithiasis

**DOI:** 10.1186/s12893-023-02222-0

**Published:** 2023-10-17

**Authors:** Bo Yuan, Xuanfeng Zhang, Chenchen Kong, Cancan Zhang, Huansong Li

**Affiliations:** 1 Center of Hepatobiliary Pancreatic Disease, XuZhou Central Hospital, Xuzhou, Jiangsu PR China; 2 Department of Gastroenterology and Endocrinology, XuZhou Central Hospital, Xuzhou, Jiangsu PR China

**Keywords:** Laparoscopic choledocholithotomy, Primary closure, Choledocholithiasis, Propensity score matching

## Abstract

**Background:**

Based on the current trend of increasing incidence of choledocholithiasis, it is of great significance to explore the closure method of the common bile duct during laparoscopic choledocholithotomy.

**Methods:**

Backtracking full-thickness continuous everting suture was selected for primary closure of the common bile duct suture, while traditional T-tube drainage was selected for the control group. Propensity score matching (PSM) was used to reduce baseline differences between the two groups.

**Result:**

The intraoperative blood loss, operation time, postoperative recovery speed, postoperative bleeding, postoperative pancreatitis, recurrence rate of bile duct stones, and hospitalization time in the primary closure group were all less than those in the T-tube drainage group.

**Conclusion:**

Under certain conditions, backtracking full-thickness continuous everting suture could benefit patients with choledocholithiasis compared with traditional T-tube drainage.

## Introduction

In recent years, more and more literature reports and clinical statistics show that the incidence of choledocholithiasis is increasing year by year. The reasons are mostly related to the increase of living standards, high-fat diet, obesity, diabetes, and genetic factors [1]. Among them, common bile duct stones (CBDS) are more harmful to human, because of acute obstructive suppurative cholangitis (AOSC) which is caused by common bile duct stones combined with acute infection [2]. There are many treatments for choledocholithiasis, such as open choledocotomy, ERCP, and laparoscopic choledocotomy. There are two widely accepted methods of suture of the common bile duct during laparoscopic choledochotomy: primary closure (PC) and biliary drainage (BD). Traditional biliary drainage has a long duration of catheterization, which reduces the quality of life of patients and brings trouble to home care. Especially in patients with poor nutritional status, there is the possibility of biliary peritonitis and septic shock caused by the shedding of the drainage tube [3]. There are also some postoperative complications in primary closure, the most common of which is postoperative bile leakage. To reduce the incidence of bile leakage, surgeons employ new suturing techniques, special sutures, and assisted biliary drainage [4]. Therefore, comparing the pros and cons of primary closure and biliary drainage is of great significance for clinical decision-making. We used a retrospective study method to compare the postoperative complication rates of novel primary closure (laparoscopic backtracking full-thickness continuous everting suture) and traditional biliary drainage in non-AOSC patients.

## Method

### Patients

All patients who underwent laparoscopic choledochotomy at Xuzhou Central Hospital between January 2016 and March 2019 were included in the preliminary screening results (*n* = 279). Of these, 94 patients had conventional T-tube biliary drainage and 185 had primary closure (backtracking full-thickness continuous everting suture).

### Patient inclusion criteria

- Preoperative abdominal ultrasonography and MRCP confirmed common bile duct stones combined with cholecystolithiasis.
- Patients without acute obstructive suppurative cholangitis.
- The patients underwent laparoscopic choledochotomy.

### Patient exclusion criteria

- Patients converted to laparotomy.
- Patients with intrahepatic bile duct stones.
- Patients with common bile duct stenosis.
- Patients with biliary tract tumors.
- Children and pregnant women.

### Ethical approval

This study is a retrospective study and has been approved by the Ethics Committee of Xuzhou Central Hospital. The clinical information and imaging data involved in the study have obtained the informed consent of all participants and/or their Legal guardian.

### The surgical procedure before suture of the common bile duct

First, remove part of the adipose tissue on the surface of the anterior wall of the common bile duct to expose the anterior wall of the common bile duct. An incision of about 1 cm in length was cut along the longitudinal axis of the common bile duct, which was appropriately adjusted according to the size of the stone. After the common bile duct stones were removed, the intrahepatic and extrahepatic bile ducts were explored in turn to confirm that there were no residual stones. The common bile duct was explored to determine the absence of edematous strictures at the lower end of the common bile duct.

### Primary closure (backtracking full-thickness continuous everting suture)

4–0 PDS II absorbable sutures were selected for common bile duct suture. Suture and knot from the cephalad of the bile duct incision, and suture the distal end with continuous full-thickness valgus suture. The stitching distance is about 3 mm, and the margin is about 1 mm. After the first layer of suture is completed, continue to return the suture to the cephalad of the common bile duct.

### T-tube biliary drainage

First, a T-tube is placed in the common bile duct. Then, interrupted full-thickness sutures of the common bile duct were performed with 4-0 PDS II absorbable sutures.

### Developing propensity score

In retrospective observational studies, there may be some imbalance in clinical and sociodemographic characteristics. And these imbalances can affect observations. Propensity score matching (PSM) can substantially reduce baseline differences between groups in observational studies [5]. To remove the effect of these imbalances on the observations, PSM was used to correct baseline data and remove confounding bias.

### Statistics

Two-sample independent t-test was used to compare groups of normally distributed data. Comparisons between groups for non-normally distributed data were performed using the two-sample Mann-Whitney U test. Logistic regression was used to screen for risk factors associated with recurrence of choledocholithiasis. Statistical analysis processes use SPSS25.0 software. *P* < 0.05 means the difference is statistically significant.

## Result

### PSM model

We constructed a cohort of 279 patients with choledocholithiasis, where 94 patients underwent BFCE suture and 185 patients underwent biliary drainage. As shown in Table 1, the age difference between the two groups of patients was statistically significant (*P* < 0.05). Age can affect the postoperative recovery of patients with choledocholithiasis. For example, the nutritional status of elderly patients is worse, and the incidence of postoperative complications is higher. After developing PSM model, 182 patients were selected for the observation cohort and all differences in clinical characteristics were not statistically significant (*P* > 0.05) (Table 1).

**Table 1:** Clinical characteristics of the two groups of patients before and after Propensity score matching

Characteristics	Before PSM				After PSM			
	BFCE suture (*n* = 94)	T-tube drainage (*n* = 185)	χ^2^/Z	*P*	BFCE suture (*n* = 91)	T-tube drainage (*n* = 91)	χ^2^/Z	*P*
Gender [n(%)]			4.296	0.038			0.09	0.765
Male	54(57.4)	82(44.3)			53(58.2)	51(56.0)		
Female	40(42.6)	103(55.7)			38(41.8)	40(44.0)		
Age [*M(P25,P75)*]	64(54,72)	56(50.5,68)	-2.725	0.006	64(54,72)	65(53,73)	-0.415	0.678
Preoperative Tbil [umol/L,*M(P25,P75)*]	41.4(35.2,51.9)	43.7(32.9,54.8)	-0.963	0.335	42.1(35.2,51.9)	43.1(31.6,53.9)	-0.31	0.757
Preoperative hospital stay [d,*M(P25,P75)*]	5(3,7)	5(3,8)	-0.168	0.867	5(3,7)	4(2,7)	-1.578	0.115
BMI[*M(P25,P75)*]	26.8(22.5,30.8)	27.9(22.6,31.5)	-0.879	0.379	27(22.5,30.8)	27.8(22.4,31.2)	-0.256	0.798
ALB [g/L,*M(P25,P75)*]	34.2(31.0,36.1)	33.9(31.3,35.5)	-0.533	0.594	34.2(31.1,36.1)	32.7(30.6,35.0)	-1.053	0.292
Common bile duct diameter [mm,*M(P25,P75)*]	15.0(11.8,18.0)	14.5(12.2,17.4)	-0.664	0.507	15(11.8,18.0)	14.5(12.4,17.5)	-0.391	0.696
Hypertension [n(%)]			1.085	0.298			0.034	0.854
Positive	20(21.3)	30(16.2)			19(20.9)	18(19.8)		
Negative	74(78.7)	155(83.8)			72(79.1)	73(80.2)		
Coronary heart disease [n(%)]			1.071	0.301			1.72	0.189
Positive	10(10.6)	28(15.1)			9(9.9)	15(16.5)		
Negative	84(89.4)	157(84.9)			82(90.1)	76(83.5)		
Type 2 diabetes [n(%)]			0.393	0.531			0.054	0.817
Positive	12(12.8)	19(10.3)			11(12.1)	10(11.0)		
Negative	82(87.2)	166(89.7)			80(87.9)	81(89.0)		
Cerebral infarction [n(%)]			0.179	0.672			0.028	0.867
Positive	24(25.5)	43(23.2)			24(26.4)	25(27.5)		
Negative	70(74.5)	142(76.8)			67(73.6)	66(72.5)		
COPD [n(%)]			2.737	0.098			0.871	0.351
Positive	8(8.5)	7(3.8)			7(7.7)	4(4.4)		
Negative	86(91.5)	178(96.2)			84(92.3)	87(95.6)		
History of upper abdominal surgery [n(%)]			0.777	0.378			2.643	0.104
Positive	17(18.1)	26(14.1)			15(16.5)	24(26.4)		
Negative	77(81.9)	159(85.9)			76(83.5)	67(73.6)		

### Intraoperative clinical features

Observing the statistical indicators during the operation, it was found that the bleeding volume and operation time of the patients in the BFCE suture group were less than those in the T-tube drainage group (*P* < 0.05) (Table 2). However, there was no significant difference between the two groups in the bile duct sediment-like stones found in intraoperative exploration.

**Table 2:** Comparison of clinical efficacy between two groups

Characteristics	BFCE suture (*n* = 91)	T-tube drainage (*n* = 91)	χ^2^/Z	*P*
Intraoperative blood loss	20(20,25)	60(50,80)	-10.529	< 0.000
Operation time [min,*M(P25,P75)*]	120(100,140)	120(110,140)	-2.202	0.028
Intestinal exhaust time [h,*M(P25,P75)*]	13(11,16)	22(20,26)	-10.685	< 0.000
First activity time [h,*M(P25,P75)*]	13(11,15)	22(19,24)	-10.99	< 0.000
Postoperative Tbil [umol/L,*M(P25,P75)*]	20.3(16.1,23.6)	19.8(16.4,23.7)	0.124	0.901
Drainage tube removal time [h,*M(P25,P75)*]	88(79,97)	87(80,96)	-0.073	0.942
Postoperative return to work [h,*M(P25,P75)*]	16(12,19)	58(50,64)	-11.526	< 0.000
Postoperative blood loss > 300 ml [n(%)]				
Positive	7(7.7)	21(23.1)		
Negative	84(92.3)	70(76.9)	8.273	0.004
Postoperative bile leakage [n(%)]				
Positive	7(7.7)	9(9.9)		
Negative	84(92.3)	82(90.1)	0.274	0.601
Pancreatitis [n(%)]				
Positive	5(5.5)	15(16.5)		
Negative	86(94.5)	76(83.5)	5.617	0.018
Bile duct sediment-like stones [n(%)]				
Positive	26(28.6)	37(40.7)		
Negative	65(71.4)	54(59.3)	2.937	0.087
Choledocholithiasis recurrence [n(%)]				
Positive	10(11.0)	21(23.1)		
Negative	81(89.0)	70(76.9)	4.705	0.03
The number of days in hospital [d,*M(P25,P75)*]	16(13,19)	18(14,23)	-2.931	0.033
Hospital costs [Yuan,*M(P25,P75)*]	38,580(34,140,44,818)	44,081(34,836,54,092)	-2.272	0.013

### Postoperative paraclinical characteristics

Patients in the BFCE suture group had a shorter recovery time. The first activity time, intestinal exhaust time, and hospital stay of the patients in the BFCE suture group were shorter than those in the T-tube drainage group (*P* < 0.05) (Table 2). The postoperative total bilirubin level and the duration until the drainage tube was removed did not significantly vary between the two groups.

### Postoperative applications

The incidence of postoperative bleeding and acute pancreatitis in the BFCE suture group was significantly lower than that in the T-tube drainage group (*P* < 0.05) (Table 2). Between the two patient groups, there was no discernible difference in the frequency of postoperative bile leakage.

### Socioeconomic benefits

The hospitalization time and treatment cost of the patients in the BFCE suture group were significantly less than those in the T-tube drainage group (Table 2).

### Postoperative common bile duct stone recurrence

After 1-year follow-up, 31 cases of recurrence of common bile duct stones were found. Among them, the recurrence rate of stones in the BFCE suture group was 11.0% (10/91), and the T tube drainage group was 28.6% (21/91), with a total incidence rate of 17.0% (31/182). This result showed that the recurrence rate of stones within 1 year in the BFCE suture group was significantly lower than that in the T-tube drainage group (*P* < 0.05) (Table 2).

### Unplanned admission

A 75-year-old female patient developed severe biliary peritonitis after violently pulling out the T-tube on the 8th day after discharge. She was admitted to the hospital for ultrasound-guided paracentesis, and was discharged after endoscopic nasobiliary drainage (ENBD).

### Risk factors for biliary stone recurrence

Univariate analysis of clinical features was performed in order to identify risk factors associated with the recurrence of bile duct stones. As shown in Table 3, BMI, cerebral infarction, history of upper abdominal surgery, common bile duct diameter, common bile duct sand-like stones, Pinaverium bromide, and choice of surgical procedure were associated with the recurrence of common bile duct stones (*P* < 0.05).

**Table 3:** Univariate analysis of postoperative biliary stone recurrence

Characteristics	Recurrence (*n* = 31)	No-recurrence (*n* = 151)	χ^2^	*P*
Gender [n(%)]				
Male	17 (54.8)	87 (57.6)		
Female	14 (45.2)	64 (42.4)	0.081	0.776
Age [n(%)]				
≥ 65	19 (61.3)	66 (43.7)		
< 65	12 (38.7)	85 (56.3)	3.194	0.074
Preoperative Tbil [umol/L,n(%)]				
≥ 43	20 (64.5)	70 (46.4)		
< 43	11 (35.5)	81 (53.6)	3.393	0.065
BMI				
≥ 24	27 (87.1)	85 (56.3)		
< 24	4 (12.9)	66 (43.7)	10.312	0.001
ALB [g/L,n(%)]				
≥ 33	18 (58.1)	94 (62.3)		
< 33	13 (41.9)	57 (37.7)	0.191	0.662
Hypertension [n(%)]				
Positive	9 (29.0)	28 (18.5)		
Negative	22 (71.0)	123 (81.5)	1.747	0.186
Coronary heart disease [n(%)]				
Positive	5 (16.1)	19 (12.6)		
Negative	26 (83.9)	132 (87.4)	0.058	0.81
Type 2 diabetes [n(%)]				
Positive	2 (6.5)	19 (12.6)		
Negative	29 (93.5)	132 (87.4)	0.442	0.506
Cerebral infarction [n(%)]				
Positive	14 (45.2)	35 (23.2)		
Negative	17 (54.8)	116 (76.8)	6.317	0.012
COPD[n(%)]				
Positive	0 (0)	11 (7.3)		
Negative	31 (100)	140 (92.7)	1.292	0.256
History of upper abdominal surgery [n(%)]				
Positive	13 (41.9)	26 (17.2)		
Negative	18 (58.1)	125 (82.8)	9.333	0.002
Diameter of common bile duct [mm,n(%)]				
≥ 15	23 (74.2)	61 (40.4)		
< 15	8 (25.8)	90 (59.6)	11.821	0.001
Postoperative blood loss > 300 ml [n(%)]				
Positive	5 (16.1)	23 (15.2)		
Negative	26 (83.9)	128 (84.8)	< 0.001	> 0.999
Postoperative bile leakage [n(%)]				
Positive	3 (9.7)	13 (8.6)		
Negative	28 (90.3)	138 (91.4)	< 0.001	> 0.999
Pancreatitis [n(%)]				
Positive	3 (9.7)	17 (11.3)		
Negative	28 (90.3)	134 (88.7)	< 0.001	> 0.999
Complication [n(%)]				
Positive	6 (19.4)	30 (19.9)		
Negative	25 (80.6)	121 (80.1)	0.004	0.948
Bile duct sediment-like stones [n(%)]				
Positive	26 (83.9)	37 (24.5)		
Negative	5 (16.1)	114 (75.5)	40.052	< 0.001
Pinaverium bromide (Oral administration) [n(%)]				
Positive	7 (22.6)	78 (51.7)		
Negative	24 (77.4)	73 (48.3)	8.735	0.003
Operation [n(%)]				
Backtracking full-thickness continuous everting (BFCE) suture	9 (29)	81 (53.6)		
T-tube drainage	22 (71)	70 (46.4)	6.232	0.013

To further analyze the risk factors for postoperative gallstone recurrence, a multivariate analysis represented by logistic regression analysis was performed. The results of analysis showed that Age (OR = 3.497, *P* = 0.027), Preoperative Tbil (OR = 3.908, *P* = 0.015), BMI (OR = 4.45, *P* = 0.027), History of upper abdominal surgery (OR = 5.38, *P* = 0.005), Bile duct sediment-like stones (OR = 24.849, *P* < 0.001), and Pinaverium bromide (OR = 0.226, *P* = 0.012) were risk factors for recurrence of common bile duct stones (Table 4).

**Table 4:** Logistic regression analysis of postoperative biliary stone recurrence

Characteristics	b value	Standard deviation	Wald value	*P*	OR	95% CI
Age ≥ 65	1.252	0.564	4.923	0.027	3.497	1.157~10.565
Preoperative Tbil ≥ 43umol/L	1.363	0.563	5.859	0.015	3.908	1.296~11.783
BMI ≥ 24	1.493	0.674	4.9	0.027	4.45	1.187~16.688
History of upper abdominal surgery	1.683	0.595	7.985	0.005	5.38	1.675~17.286
Bile duct sediment-like stones	3.213	0.651	24.32	< 0.001	24.849	6.931~89.095
Pinaverium bromide (Oral administration)	-1.488	0.591	6.345	0.012	0.226	0.071~0.719

## Discussion

Hepatobiliary surgeons are more inclined to choose laparoscopic surgery for treatment. There are two mainstream methods for the management of postoperative bile ducts: primary closure and T-tube drainage. In this study, the backtracking full-thickness continuous everting suture was chosen instead of the traditional simple interrupted suture or the simple continuous suture. The results showed that it also had high safety and patients could benefit from it.

Traditional biliary drainage has its corresponding advantages, such as fully draining the infected bile, reducing the pressure in the bile duct, thereby preventing bile leakage, and avoiding the biliary stricture caused by the primary closure of the bile duct. However, there are also disadvantages such as long time with the tube, loss of electrolytes in digestive juice, accidental prolapse of T tube, ischemic necrosis of bile duct wall caused by T tube compression, and increased cost. It has been reported in the previous study that T-tube-related complications can be as high as 15.3% after laparoscopic choledocholithotomy [6]. To our surprise, recurrence of common bile duct stones was more likely in patients who received T-tube drainage. This also again illustrates the superiority of our BFCE suture over the traditional procedure (T-tube drainage). However, this result may have been influenced by the inflammation of the biliary system at the time of our choice of BFCE closure.

For primary closure, it also has its own unique shortcomings, such as postoperative bile leakage and bile duct stricture. However, with the maturity of the technology and the improvement of the operator’s surgical operation, the incidence rate of serious complications has dropped significantly. At the same time, some researchers have demonstrated that the primary closure does not increase the postoperative complications [7]. The BFCE group had less intraoperative blood loss, shorter operation time, earlier first postoperative ambulation time, earlier intestinal ventilation recovery time, and shorter hospital stay, which was in line with the concept of fast track surgery (FTS). Despite these advantages, primary closure has limitations in terms of use. When the diameter of the common bile duct is less than 8 mm, the gallbladder inflammation and adhesion are serious, and the bile duct wall is thin, it is no longer meaningful to insist on primary closure, which will inevitably bring serious complications. T-tube drainage is still a safe measure in special circumstances [8].

For patients with primary closure, the most common complication was postoperative bile leakage. Many experts and scholars have also used a variety of methods to try to improve it to reduce the occurrence of bile leakage after primary closure. For example, (1) Improvement of suture methods; (2) Change of suture material [9]; (3) Biliary stent, C tube, J tube and nasobiliary drainage were placed during the operation [10, 11]. In this study, 16 patients (8.8%, 16/182) developed bile leakage after operation in the two groups. According to the classification of bile leakage provided by the International Study Group of Liver Surgery in 2011 [12], there were 12 cases of grade A and 4 cases of grade B. All of them were relatively mild bile leakage. After conservative treatment and appropriate extension of drainage tube removal time, all of them recovered within 6 days. The incidence of bile leakage was 7.7% (7/91) in the BFCE group and 9.9% (9/91) in the T-tube drainage group. There was no significant difference between the two groups (*P* > 0.05).

The recurrence of gallstones, including common bile duct stones and intrahepatic bile duct stones, has been an important factor affecting the long-term quality of life of patients. Previous studies have suggested that the recurrence of choledocholithiasis is related to age, common bile duct diameter, metabolism-related diseases, and treatment methods [13]. In our study, age, BMI, preoperative total bilirubin level, history of upper abdominal surgery, and common bile duct sand-like stones were identified as risk factors for stone recurrence. Different from the previously proven risk factors, a history of upper abdominal surgery and common bile duct sand-like stones were newly identified risk factors. We hypothesized that upper abdominal surgery caused abdominal adhesions, which affected the common bile duct and changed the velocity of bile flow. In addition, univariate analysis showed that different surgical methods also affected the recurrence of common bile duct stones. This also provides a strong basis for us to prioritize BCFE in surgical methods. We believe that it is precisely because the primary closure avoids the implantation of T-tube and does not affect the flow of bile in the common bile duct after operation, so that it maintains a sufficient flow rate and avoids the recurrence of stones caused by cholestasis.

This is a retrospective study, which proves that under certain conditions, the choice of backtracking full-thickness continuous everting suture is safe for the treatment of common bile duct stones. But there are limitations to our study. Further in vitro and in vivo studies are needed to demonstrate the association between Pinaverium bromide and choledocholithiasis recurrence. The number of cases included in the study is still small, and further randomized controlled trials will confirm our conclusions more rigorously.

## Conclusion

In this study, we showed that primary closure (backtracking full-thickness continuous everting suture) reduced operative time, intraoperative blood loss, hospitalization costs, and postoperative complication rates in patients with choledocholithiasis. Compared with traditional T-tube biliary drainage, backtracking full-thickness continuous everting suture is an alternative and better new biliary suture method.

## Data Availability

The datasets created and analyzed for the current investigation are not publically accessible since it would be inconvenient to disclose patient privacy; however, they are available from the corresponding author upon justifiable request (Xuanfeng Zhang: zxfujs@126.com).
